# CD177 Expression and Inflammation Grade in Helicobacter pylori-Infected Wild-Type and CD177^−/−^ C57BL/6 Mice

**DOI:** 10.1155/2019/9506863

**Published:** 2019-04-04

**Authors:** Xing-Tang Yang, Zhi-Jun Wang

**Affiliations:** Department of Emergency, Tenth People's Hospital of Tongji University, Shanghai 200072, China

## Abstract

This study was undertaken to further investigate the CD177 expression in Helicobacter pylori- (Hp-) infected wild-type and CD177^−/−^ C57BL/6 mice, which may be helpful to elucidate the relationship between CD177 and Hp-related gastritis. 20 WT mice were randomly assigned into the Hpss1 WT group (*n* = 10) and Hp49503 WT group (*n* = 10); 20 KO mice were randomly assigned into the Hpss1 KO group (*n* = 10) and Hp49503 KO group (*n* = 10). The remaining mice served as controls. Mice in the HpSS1 groups and Hp49503 groups were independently infected with corresponding strains. Results showed that the Hp colonization score was related to the grade of mucosal inflammation (*P* < 0.05). The inflammation grade was comparable between the HpSS1 group and Hp49503 group as well as between the WT group and KO group. In addition, the Hp colonization score was related to the CD177 expression score (*P* < 0.05). The CD177 expression in the Hp colonization group was higher than that in the non-Hp colonization group (*P* < 0.05). CD177 expression was positively related to the inflammation grade (*P* < 0.01). In conclusion, CD177 expression was similar between HP49503- and HPss1-infected WT C57BL/6 mice, and CD177 expression was undetectable in CD177^−/−^ mice. CD177 expression in the gastric mucosa increases with the elevation of inflammation grade. In Hp-infected mice, the inflammation grade had no relationship with the type of Hp strain and the CD177 expression, but the mucosal inflammation score in Hp-infected mice was higher than that in non-Hp infected mice.

## 1. Introduction

Helicobacter pylori (Hp) is a type of gram-negative microaerophile and mainly found in the gastric mucosa of humans and primates. About half of the population is infected by Hp [1, 2]. Hp infection is an important pathogenic factor of gastritis and peptic ulcer. The World Health Organization has classified Hp as the class I pathogenic factor, and it is closely related to the pathogenesis of gastric mucosa-associated tissue lymphoma. Thus, increasing attention has been paid to the Hp eradication, but the abuse or irrational use of antibiotics significantly increases the drug resistance of Hp [3–7]. Thus, the Hp eradication and the pathogenesis of Hp infection have become focuses in the field of gastroenterology. CD177 is a member of the leukocyte antigen 6 superfamily and can encode two neutrophil-related proteins: NB1 and PRV-1 [5, 8]. There is evidence showing that CD177 expression increases on neutrophils as a response to inflammation [9]. In recent years, some studies have revealed that CD177 is closely related to some diseases due to inflammation dysregulation, such as polycythemia vera and thrombocytopenia. In addition, CD177 has been a diagnostic marker for some diseases such as polycythemia vera, thrombocytopenia, and idiopathic myelofibrosis [5, 10–12].

In our previous study, results showed that CD177 expression in the gastric mucosa of patients with Hp-related gastritis was significantly higher than that of patients with non-Hp related gastritis [13]. Our group has successfully established an infection model with CD177^−/−^ mice. This study was undertaken to further investigate the CD177 expression in CD177^−/−^ mice, aiming at elucidating the relationship between CD177 expression and Hp-related gastritis.

## 2. Materials and Methods

### 2.1. Materials

Sydney strain 1, H. pylori SS1 (VacA+, CagA-), and Hp49503 (VacA+, CagA+) strains were purchased from the Department of Pathogenic Biology, School of Medicine, Shanghai Jiao Tong University. Brucella Broth was provided by the Municipal Center for Disease Control and Prevention of Shanghai. Agar powder was purchased from Oxoid (UK). Simple culture tank with ventilation was prepared by the Shanghai Academy of Life Sciences, Chinese Academy of Sciences. Others included Gram staining kit (Shanghai Kang'en Biotech Co. Ltd), H. pylori detection kit (urease method) (Zhuhai Lituo Biotech Co. Ltd), diaminobenzidine hydrochloride (DAB) (Sigma, USA), mixed gas (85% N_2_, 10% CO_2_, 5% O_2_) (Shanghai Youjiali Liquid Nitrogen Co. Ltd.), high-pressure steam sterilizer (SANYO, Japan), ultraclean workbench (Suzhou Yongjiu Pressure Regulator Factory), CX31 microscope (Olympus, Japan), electronic balance (Sartorius, Germany), RM2145 slicer (Leica, Germany), ultralow temperature refrigerator (SANYO, Japan), CD177 antibody (ab8092; Abcam), UV inhibitor (Roche Diagnostics), UV UNIV MULT HRP (Roche Diagnostics), carbolic acid red dye solution (Beijing Leigen Biotech Co. Ltd), ultrapure water meter (Millipore, USA), electric thermostatic water bath (Shanghai Yiheng Co. Ltd), and benchtop horizontal centrifuge (Eppendorf, Germany).

### 2.2. Animals

This study was conducted according to the Guide for the Care and Use of Laboratory Animals (National Institutes of Health; No. 85.23; Revision 1996). Animal procedures were performed in accordance with the Guide for the Care and Use of Laboratory Animals of the Tenth People's Hospital. 44 C57BL/6 mice were purchased from the Experimental Animal Center of the Tenth People's Hospital of Tongji University. Animals were housed according to the standards in the levels of laboratory animal microorganisms and parasites. Specific pathogen-free healthy CD177^−/−^ C57BL/6 mice (CD177 KO; *n* = 22) and wild-type C57BL/6 mice (WT; *n* = 22) aged 6-8 weeks and weighing 18-30 g were used in this study and housed in the Experimental Animal Center of the Tenth People's Hospital of Tongji University. There were 11 males and 11 females in each group. All the animals were allowed to accommodate to the environment for 7 days and in a SPF environment with 12 h/12 h light/dark cycle. Animals were given ad libitum access to water and food, and the room temperature and humidity were controlled at 25°C and 60%, respectively. There were 5-6 animals in each cage. This study was approved by the Ethics Committee of the Tenth People's Hospital and Shanghai Science and Technology Commission.

### 2.3. Establishment of Animal Model and Grouping

20 WT mice were randomly divided into the Hpss1 WT group and Hp49503 WT group (*n* = 10 per group). In addition, 20 KO mice were randomly assigned into the Hpss1 KO group and Hp49503 KO group (*n* = 10 per group). The remaining two WT and two KO mice served as WT blank controls (*n* = 2) and KO blank controls (*n* = 2), respectively.

### 2.4. Preparation of Hp Suspension

The standard Hp strains were collected and then identified by urease and oxidase detection and Gram staining. Then, the Hp strains were resuspended in PBS and the concentration of Hp was determined by spectrophotometry. When the absorbance at 660 nm was 1, the Hp concentration was 1 × 10^8^ colony-forming unit (CFU)/mL. Hp suspension at 1 × 10^8^ CFU/mL was used in the following experiments. The preparation of culture medium for Hp strains was reported in our previous studies [14–16].

### 2.5. Establishment of the Animal Model

According to previously reported [17–21], each mouse was intragastrically administered with 0.3 mL of Hp suspension at 1 × 10^8^ CFU/mL once every other day and inoculation was done thrice. In controls, mice were intragastrically administered with 0.3 mL of sterilized PBS. All the animals were deprived of food for 12 h and deprived of water for 4 h before intragastric administration. After inoculation of Hp suspension, animals continued to be deprived of water for 4 h and deprived of food. Mice in the blank control group did not receive any treatment.

### 2.6. Collection of Gastric Mucosa

At 2 weeks after the last administration of Hp suspension, mice were sacrificed, and the whole stomach was collected (including the gastric sinus, corpus, and duodenum). The stomach was opened along the greater curvature and the gastric mucosa was exposed. After washing in PBS, the gastric mucosa was separated [17]: half was embedded in paraffin for pathological examination and half was stored at -80°C for biological analysis.

### 2.7. Pathological Processing

Tissues were fixed in 10% formalin, dehydrated in ethanol, transparentized in xylene, embedded in paraffin, and sectioned. Then, sections were heated at 56°C for 2 h.

### 2.8. Routine HE Staining

For the HE staining, sections were deparaffinized, dehydrated in ethanol, and then subjected to hematoxylin staining. Following treatment with hydrochloric acid in ethanol, sections were stained with eosin for 5 min. After routine dehydration, transparentization, and mounting, sections were observed under a light microscope. The nucleus was blue and the cytoplasm and Hp were light red.

### 2.9. Carbolic Acid-Azaleine Staining

Paraffinized sections were deparaffinized and hydrated. Sections were incubated with carbolic acid-azaleine staining buffer for 3-5 min. Then, sections were washed with flowing water. After treatment with hydrochloric acid in ethanol, sections were washed with flowing water. After transparentization with xylene, sections were mounted and observed under a light microscope.

### 2.10. Immunohistochemistry for CD177

Sections (4 *μ*m) were heated at 56°C for 2 h, aiming at preventing against stripping. Sections were deparaffinized in xylene thrice (10 min for each) and then treated with absolute ethanol for 5 min, 95% ethanol for 5 min, and 75% ethanol for 5 min. Then, sections were washed with flowing water for 5 min. Sections were heated in 1x EDTA (pH = 8.0) for 10 min and then allowed to cool to room temperature and then washed in 1x PBS thrice (5 min for each). They were incubated with 0.3% hydrogen peroxide for 3 min to inactivate endogenous peroxidase and then washed in 1x PBS thrice (5 min for each). Sections were treated with CD177 antibody (100 *μ*L) at 37°C for 30 min and washed in 1x PBS thrice (5 min for each). Then, they were treated with nonspecific antigen inhibitor (UV inhibitor; Roche Diagnostics; 100 *μ*L) for 5 min and washed in 1x PBS thrice (5 min for each). Sections were incubated with UV UNIV MULT HRP (Roche Diagnostics; 100 *μ*L) at 37°C for 8 min and washed in 1x PBS thrice (5 min for each). Visualization was done with DAB (100 *μ*L) at 37°C for 4 min and washed in flowing water for 5 min. Sections were stained with hematoxylin for 5 min, then washed in flowing water, treated in ethanol containing 1% hydrochloric acid for 30 s, and incubated with warm water for 3 min, followed by washing in flowing water. Sections were treated with 75% ethanol for 5 min, 95% ethanol for 5 min, and then absolute ethanol for 5 min. Sections were allowed to dry in air, mounted with neutral gum, and then observed under a light microscope.

### 2.11. Statistical Analysis

Statistical analysis was performed with SPSS version 17.0. Comparisons of rates were done with a chi-square test. A value of *P* < 0.05 was considered statistically significant.

## 3. Results

### 3.1. Inflammation Grade and CD177 Expression of the Gastric Mucosa in Hp-Infected Mice

#### 3.1.1. HE Staining

**Figure 1:**
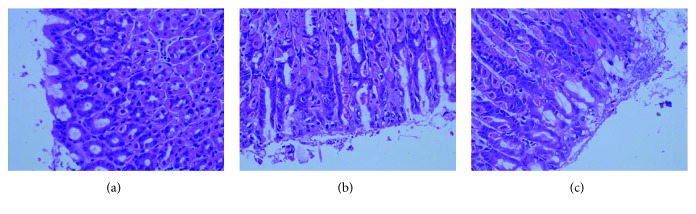
HE staining of the gastric mucosa. (a). Gastric mucosa with an inflammation grade of I and Hp colonization score of 1 in 49503KO mice. (b). Gastric mucosa with inflammation grade of II and Hp colonization score of 2 in 49503WT mice. (c). Gastric mucosa with inflammation grade of III and Hp colonization score of 3 in 49503WT mice.

HE staining showed successful Hp colonization in the gastric mucosa of mice, and inflammatory changes were also observed in the gastric mucosa. In the blank control groups, no inflammatory changes were observed in the gastric mucosa. Pathological examination indicated that the amount of Hp colonized in the gastric mucosa increased with the increase in inflammation grade. Especially, a large amount of Hp was found in the gastric mucosa of mice with an inflammation grade of III (Figures 1(a)–1(c)).

#### 3.1.2. Immunohistochemistry

**Figure 2:**
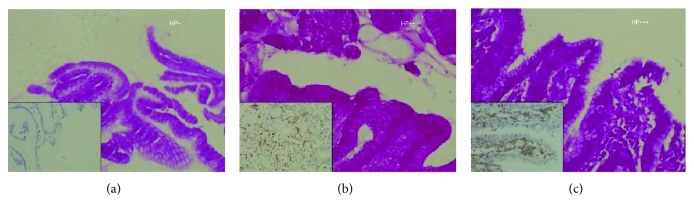
Immunohistochemistry of the gastric mucosa. (a). Gastric mucosa with inflammation grade of I, Hp colonization score of 1, and CD177 expression score of 1. (b). Gastric mucosa with inflammation grade of II, Hp colonization score of 2, and CD177 expression score of 2. (c). Gastric mucosa with inflammation grade of III, Hp colonization score of 3, and CD177 expression score of 3. Arrows indicate HP aggregation.

The Hp colonization and CD177 expression were assessed in mice with different inflammation grades (Figures 2(a)–2(c)). Azaleine staining showed that the score of Hp colonization increased with the increase in inflammation score; in addition, immunohistochemistry showed the CD177 expression increased with the increase in inflammation score. CD177 expression increased significantly in both standard Hp strain-infected C57BL/6 WT groups, respectively. However, CD177 expression was similar between them. And there was no CD177 expression in KO mice.

### 3.2. Correlation between Hp Colonization Score and Inflammation Grade

The amount of Hp colonized was comparable between mice with inflammation grades of I and II (*P* > 0.05). Significant difference was observed in the amount of Hp colonized between mice with inflammation grades of III and I: the Hp colonization rate in inflammation grade of III was better than that in inflammation grade of I (*P* < 0.05). There was no significant difference in the amount of Hp colonized between mice with inflammation grades of III and II (*P* > 0.05) (Table 1).

### 3.3. Inflammation Grade of KO Mice and WT Mice Inoculated with Hpss1 Strain and Hp49503 Stain

There was no significant difference in the inflammation grade among groups (*P* > 0.05): significant difference was not observed between KO mice and WT mice as well as between mice treated with the Hpss1 strain and those treated with the Hp49503 strain (Table 2).

### 3.4. Correlation between Hp Colonization and CD177 Expression

Positive CD177 expression was observed in mice with and without Hp colonization. There was significant difference in the distribution of CD177 expression among mice with different Hp colonization scores (*P* < 0.05). The CD177 expression was markedly different between mice with Hp colonization score of 3 and those without Hp colonization (*P* < 0.05). The CD177 expression was similar among mice with different Hp colonization scores (*P* > 0.05) (Table 3).

### 3.5. Correlation between CD177 Expression and Inflammation

CD177 expression had a close relationship with inflammation grade (*P* ≤ 0.001). Intragroup paired comparisons showed that the CD177 expression was similar between inflammation grade I and inflammation grade II (*P* > 0.05), but there was marked difference between inflammation grade I and inflammation grade III (*P* < 0.01) as well as between inflammation grade II and inflammation grade II (*P* < 0.01) (Table 4).

### 3.6. CD177 Expression in Mice with Hp Inoculation

There was no marked difference in the CD177 expression among WT mice with Hp inoculation (*P* > 0.05) (Table 5).

## 4. Discussion

Hp is a type of gram-negative microaerophile, and more than half of the people worldwide is affected by Hp. Hp infection has been an important pathogenic factor of some digestive diseases such as chronic gastritis, peptic ulcer, gastric cancer, and gastric mucosa-associated lymphoid tissue lymphoma [22–26]. CD177 is a member of leukocyte antigen 6 superfamily and encodes two neutrophil-related proteins: NB1 and PRV-1. It has been found that CD177 expression increases significantly on neutrophils as a response to inflammation [5, 8, 9]. In recent years, studies reveal that CD177 is related to some diseases including polycythemia vera and thrombocytopenia, and CD177 has been a diagnostic marker of some diseases (such as polycythemia vera, thrombocytopenia, and idiopathic myelofibrosis) [5, 10–12]. However, little is known about the role of CD177 in the Hp-related gastritis.

Our study showed that the CD177 expression was significantly higher in Hp-related gastritis than in non-Hp related gastritis [13]. In addition, we had successfully established CD177 knockout mice which were infected by Hp in our previous study. In the present study, the CD177 expression was further detected in CD177 KO mice, aiming at elucidating the relationship between CD177 expression and Hp-related gastritis.

In the present study, pathological examination showed that the Hp colonization increased with the elevation of the inflammation grade of the gastric mucosa. Especially, a large amount of Hp was found in the gastric mucosa with the inflammation grade of III. This was further confirmed in following examinations.

Then, the correlation between Hp colonization and inflammation grade of the gastric mucosa was further assessed. Results showed that Hp colonization had a close relationship with the inflammation grade, and the inflammation grade of the gastric mucosa with successful Hp colonization was better than that of the mucosa without Hp colonization. However, among mice with Hp colonization, the Hp colonization score was not related to the inflammation grade, which might be related to the differences in the Hp strains, type of mice, or the small sample size. In several mice with Hp inoculation, inflammation was still observed although successful Hp colonization was not observed, but inflammation was not found in mice of the blank control group. This indicates that the gastric mucosal inflammation in mice without Hp colonization might be related to immune-related clearance of Hp, but the specific mechanism is warrant to a further study. The inflammation grade was further compared among KO mice and WT mice with inoculation of Hpss1 and Hp49503. Results showed that the inflammation grade was not related to the strain of Hp in both KO mice and WT mice: in both KO and WT mice, the inflammation grade was comparable between mice inoculated with Hpss1 and Hp49503; in mice inoculated with Hpss1 and Hp49503, the inflammation grade was similar between KO mice and WT mice.

The relationship between Hp colonization score and CD177 expression was further assessed. Results showed that the colonization score was closely related to CD177 expression. The CD177 expression score in mice with Hp colonization was better than that in those without Hp colonization. The relationship between CD177 and inflammation grade was further evaluated. Results showed that the CD177 expression score was closely related to the inflammation grade, and the CD177 expression score increased with the increase in inflammation grade. The CD177 expression was also compared among mice with Hp inoculation, and results showed no CD177 expression in KO mice inoculated with Hp49503 or Hpss1; the CD177 expression was similar between WT mice inoculated with Hp49503 and Hpss1.

Taken together, the CD177 expression is similar between WT mice inoculated with Hp49503 and Hpss1. CD177 expression was not observed in KO mice. CD177 expression score has a direct correlation with inflammation grade, and CD177 expression increases with the elevation of inflammation grade. The inflammation grade is related to Hp colonization, and the inflammation grade in mice with Hp colonization is better than that in mice without Hp colonization. However, the inflammation grade has no relationship with the strain of Hp and the type of mice (WT or KO). In several mice without Hp colonization, inflammation is also present in the gastric mucosa, which might be related to the factors other than Hp colonization (strain of Hp, type of mice, sample size, or inflammation-related cytokines), but the specific mechanism should be further studied. In addition, the relationship of the CD177 expression score with some cytokines and receptors in WT C57BL/6 mice should be further elucidated in more studies.

## Figures and Tables

**Table 1:** Hp colonization score and inflammation grade.

Hp colonization score	Inflammation grade			*P*
	I	II	III	
0	2	0	0	0.001
1	1	2	2	
2	2	6	8	
3	0	2	15	

**Table 2:** Inflammation grade of KO mice and WT mice inoculated with Hpss1 strain and Hp49503 stain.

Inflammation grade	Hp strains				*P*
	Hp49503		HpSS1		
	KO (*n* = 10)	WT (*n* = 10)	KO (*n* = 10)	WT (*n* = 10)	
I	2	2	1	0	0.469
II	2	4	1	3	
III	6	4	8	7	

**Table 3:** Correlation between Hp colonization and CD177 expression.

Hp colonization score	CD177 expression score (*n* = 20)			*P*
	1	2	3	
0	1	0	0	0.047
1	1	2	1	
2	1	3	2	
3	0	1	8	

**Table 4:** Correlation between CD177 expression and inflammation.

Inflammation grade	CD177 expression score (*n* = 20)				*P*
	0	1	2	3	
I	0	2	0	0	*P* ≤ 0.001
II	0	1	5	1	
III	0	0	1	10	

**Table 5:** CD177 expression in mice with Hp inoculation.

Group	CD177 expression score (*n* = 20)				*P*
	0	1	2	3	
Hp49503 WT	0	2	2	6	0.587
Hpss1 WT	0	2	4	4	

## Data Availability

The data used to support the findings of this study are available from the corresponding author upon request.
